# The Pocket-Handkerchief—A Seed-Sack of Death

**Published:** 1919-04-05

**Authors:** 


### The Pocket-handkerchief—A Seed-Sack of Death.

It is a remarkable thing that during the recent great "offensives" of the influenza so little attention was paid to the pocket-handkerchief as a source of infection. In the organic substance of an ordinary handkerchief and in the organic catarrhal discharge on it germs can feed and thrive and multiply, so long as the handkerchief is moist, while as soon as it is dry every flourish shakes infectious dust into the air. A dirty handkerchief used—it may be for several days—by an influenza patient is covered with decomposing organic material, and kept at a steady temperature in a warm pocket, is an admirable hot-bed and incubator for the intensive cultivation of microbes.

The public were told to douche their noses and to gargle their throats. They were told to use masks, which are probably as useful as herring-nets, to intercept germs either coming or going—which interfere with free breathing and which render the air inspired unhealthily warm and wet. They were told to avoid crowds, which were probably unavoidable, and they were given other advice, good, bad and indifferent, and yet nothing was said about that seed-sack of death the pocket-handkerchief, and no doubt it went on disseminating disease in all directions, for almost no attempt was made to sterilise handkerchiefs in the wash or to guard against the infection of other handkerchiefs. In the circumstances laundry workers were specially subject to the "'flu," and no wonder.

If future epidemics of influenza are to be checked not only eating utensils but handkerchiefs as sources of infection must be carefully sterilised, not merely perfunctorily washed in water more or less hot. At sanatoria for consumptives the handkerchiefs used by patients are always soaked in disinfectant before being washed, and during epidemics this should be made a general rule. Further, the public must be taught that handkerchiefs, like bandages, should be changed as soon as they are soiled, and that a dirty handkerchief is not only æsthetically objectionable but a source of danger both to the owner and to the community.

It must be noted, too, that under certain conditions the pocket itself is liable to become septic and infectious. The writer has demonstrated microbes in the handkerchief pockets of consumptives. So that in times of pandemic and epidemic it will be a good thing to sprinkle both handkerchief and pocket with some volatile disinfectant in addition to taking further measures thoroughly to sterilise both. And if people can be persuaded to use paper handkerchiefs and to burn them as soon as soiled that would be a still more effective means of combating infection. Whatever practical measures be taken for the purpose, it is certainly necessary to pay more attention to the sterilisation of pocket-handkerchiefs.

